# Editorial: Exercise-induced oxidative stress and the role of antioxidants in sport and exercise

**DOI:** 10.3389/fspor.2023.1269826

**Published:** 2023-08-15

**Authors:** Hannah J. Moir, Marcin Maciejczyk, Mateusz Maciejczyk, Felipe J. Aidar, Hamid Arazi

**Affiliations:** ^1^ School of Life Sciences, Pharmacy and Chemistry, Faculty of Health, Science, Social Care and Education, Kingston University, London, United Kingdom; ^2^ EMJ, London, United Kingdom; ^3^ Faculty of Physical Education and Sport, University School of Physical Education, Kraków, Poland; ^4^ Department of Hygiene, Epidemiology and Ergonomics, Medical University of Bialystok, Białystok, Poland; ^5^ Department of Physical Education, Federal University of Sergipe, São Cristóvão, Brazil; ^6^ Department of Exercise Physiology, Faculty of Sport Sciences, University of Guilan, Rasht, Iran

**Keywords:** oxidative stress, exercise-induced oxidative damage, fatigue, high-intensity (strenous) exercise, redox, antioxidants

**Editorial on the Research Topic** Exercise-induced oxidative stress and the role of antioxidants in sport and exercise

## Introduction

Oxidative stress in the human body results from an imbalance between the antioxidant system and the generation of reactive oxygen (ROS) and nitrogen species (RNS) (1). The consequence of high disturbances of redox homeostasis is impaired intra- and intercellular signalling pathways controlled by redox processes, as well as damage to molecules induced by the overproduction of ROS and RNS (2). These oxidative stress markers are generated in muscle fibres during rest, and their production is increased during muscle contraction. Exercise-induced oxidative stress could be detrimental to health and is associated with oxidant damage to macromolecular structures and accelerated muscular fatigue (2, 3).

Antioxidative enzymes serve as an important antioxidant defence line against such oxidative stress (4). Conversely, the exercise-induced overproduction of ROS in skeletal muscle can also play a vital role in skeletal muscle adaptations to different types of high-intensity exercise training, particularly endurance training (5). The extent of disruption in redox homeostasis depends on, among other factors, the duration and intensity of the effort, the level of antioxidants, and the nutritional, and physiological status of the individual, and there may be sex differences between males and females in this regard (6–8).

The aim of this Research Topic is to provide an article collection that enhances scientific knowledge and provides insights into exercise-induced oxidative stress and the role of antioxidant supplementation in sport and exercise. The current issue comprises a selection of papers covering topics such as diet and nutritional supplements for managing exercise-induced damage and oxidative stress, as well as exercise training and interventions to combat fatigue.

The contributions originate from several fields within Exercise Physiology and Sport and Exercise Nutrition, encompassing diverse perspectives. All the articles in this Research Topic are centered around one or more of the aforementioned research areas, as evidenced below.

## Nutritional strategies for mitigating exercise-induced damage and oxidative stress

The contributions in this section highlight the potential benefits of appropriate dietary interventions to support athletic performance and overall health (9, 10).

**Zare et al**. investigated the link between male football players’ diet and their oxidative biomarkers, finding that football players had lower excretion of 8-hydroxy-2-deoxyguanosine (8-OHdG) and F2alpha-isoprostane (F2a-IP) and dietary inflammatory index, as well as higher dietary total antioxidant capacity and dietary phytochemical index (*P* < 0.001) compared to non-athlete controls. These dietary indices were associated with oxidative biomarkers, indicating the importance of a healthy diet in reducing oxidative stress, and suggesting that an anti-inflammatory, antioxidant-rich diet may help reduce oxidative stress in athletes. Further research is needed to elucidate the underlying mechanisms.

**Zhang et al.** explored the potential role of Pterostilbene (PTE), a phenolic compound derived from blueberries and grapes, in protecting the intestinal epithelial barrier during high-intensity exercise. Their findings in mouse running model showed that 100 mg/kg/day of PTE significantly attenuated the loss of the intestinal epithelial barrier induced by exercise for up to 12 h. *In vitro,* PTE promoted the expression of intestinal epithelial tight junction (TJ) molecules. Additionally, the authors identified that the exercise led to an abundance of gut bacterium *Alistipesis*, which is associated with lipopolysaccharide (LPS) production which was not reversed by the PTE. This study highlights the potential of PTE as a possible nutritional supplement for preserving the integrity of the intestinal epithelial barrier, which may have protective effects on gastrointestinal health in individuals engaging in high-intensity exercise. Further research is warranted to extrapolate these findings to a human athletic population.

## Combatting fatigue: exercise training and interventions

High-intensity exercise can induce fatigue, potentially due to an excess of ROS, leading to reduced functions and increased injury risk (11).

**Silva-Reis et al.** investigated the effects of a 12-week combined aerobic and resistance training programme on lung function and mechanics and markers of airway fibrosis in obese females. The study demonstrated beneficial effects on lung function and mechanics, with improved forced vital capacity, and peak expiratory flow, with improvements in airway resistance in all groups (non-obese, obese, and obese Grade I females). The authors also observed reduced pro-fibrotic insulin-like growth factor 1 (IGF-1) and increased anti-fibrotic Klotho levels in those overweight or obese. These findings indicate the potential benefits of combined physical exercise in improving respiratory health in those overweight and obese by reducing fibrotic processes in the lungs.

Molecular hydrogen (H_2_), known for its antioxidant and anti-inflammatory properties (12), has been suggested as a potential strategy to alleviate fatigue and improve aerobic capacity (13), but its effects have not been fully characterised.

A study by **Hong et al.** demonstrated the effects of inhaling H_2_ gas before high-intensity cycling on physical fatigue and prefrontal cortex activation. They found that inhaling H_2_ gas (21.57% oxygen and 4.08% H_2_) for 20 min at 1,800 ml/min prior to exercise, reduced ratings of perceived exertion and heart rate during cycling, indicating reduced physical fatigue. Additionally, prefrontal cortex activation was maintained at high-intensity exercise (75% and 100% maximum workload). The study highlights how H_2_ gas inhalation could potentially enhance exercise performance and reduce fatigue in athletes. However, further studies are required to understand the different exercise protocols and establish an understanding of the mechanisms involved.

Finally, **Hong et al.** conducted a systematic review and meta-analysis on the effects of H_2_ intake on fatigue and aerobic capacity in healthy adults. The meta-analysis included 19 studies utilising H_2_ supplementation. Pooled effect sizes demonstrated a small significant effect on perceived exertion and blood lactate, but no impact on aerobic capacity (VO_2max_, VO_2peak_) was identified. The findings provide moderate evidence that H_2_ supplementation may alleviate fatigue in healthy adults but does not enhance aerobic capacity. The effects of H_2_ on fatigue may be influenced by factors such as training status, intervention period, and exercise types. These findings suggest that H_2_ supplementation may be beneficial for reducing perceived exertion and fatigue during exercise in healthy individuals. However, further investigation is required to determine the dose-response and impact on injury risk over time.

## Perspectives

In conclusion, this Research Topic offers insights into the role of diet and nutritional supplements in managing exercise-induced damage and oxidative stress, supporting overall health and athletic performance. Pterostilbene may have protective effects for the intestinal epithelial barrier during high-intensity exercise. Combined aerobic and resistance training can improve lung function, mechanics, and immune response, benefiting overweight and obese individuals. Hydrogen gas supplementation may alleviate fatigue in healthy adults, but it does not appear to enhance aerobic capacity. Further investigation is needed to understand the impact and mechanisms of these interventions on exercise performance and injury risk. The contributions in this Research Topic contribute to the growing body of knowledge on exercise-induced oxidative stress and its management, offering valuable insights for athletes, coaches, and researchers in the fields of Exercise Physiology and Sport and Exercise Nutrition.
